# Hydraulic Activation of the AsLOV2 photoreceptor

**DOI:** 10.1101/2025.06.19.660617

**Published:** 2025-06-25

**Authors:** Shiny Maity, Jackson Sheppard, Hannah Russell, Chungta Han, Leah Epstein, Bruce A. Johnson, Brad Price, Ruixian Han, Lokeswara Rao Potnuru, Jinlei Cui, Mark S. Sherwin, Joan-Emma Shea, Janet E. Lovett, Kevin H. Gardner, Songi Han

**Affiliations:** 1. Department of Chemistry, Northwestern University, Evanston, IL 60208, USA; 2. Department of Chemistry and Biochemistry, University of California, Santa Barbara, CA 93106, USA; 3. SUPA School of Physics and Astronomy and BSRC, University of St Andrews, North Haugh, St Andrews, KY16 9SS, UK; 4. Structural Biology Initiative, CUNY Advanced Science Research Center, New York, NY 10031, USA; 5. Ph.D. Program in Biochemistry, CUNY Graduate Center, New York, NY 10016, USA; 6. Department of Physics, Institute for Terahertz Science and Technology, University of California Santa Barbara, Santa Barbara, CA 93106, USA; 7. Integrated Molecular Structure Education and Research Center (IMSERC), Northwestern University, Evanston, IL 60208, USA; 8. Department of Chemistry and Biochemistry, City College of New York, New York, NY 10031, USA; 9. Ph.D. Programs in Biochemistry, Biology, and Chemistry, CUNY Graduate Center, New York, NY 10016, USA

## Abstract

How proteins transduce environmental signals into mechanical motion remains a central question in biology. This study tests the hypothesis that blue light activation of AsLOV2 gives rise to concerted water movement that induce protein conformational extensions. Using electron and nuclear magnetic resonance spectroscopy, along with atomistic molecular dynamics simulations at high pressure, we find that activation, whether initiated by blue light or high pressure, is accompanied by selective expulsion of low-entropy, tetrahedrally coordinated “wrap” water from hydrophobic regions of the protein. These findings suggest that interfacial water serves as functional constituents to help reshape the protein’s free energy landscape during activation. Our study highlights hydration water as an active medium with the capacity to drive long-range conformational changes underlying protein mechanics and offers a new conceptual understanding for engineering externally controllable protein actuators for biomedical studies to smart materials.

## Graphical Abstract

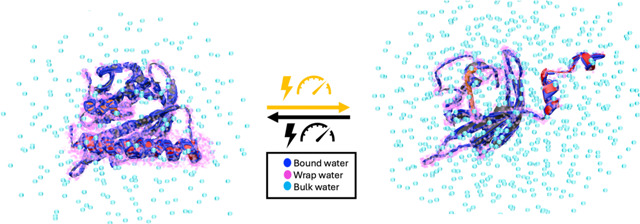

## Introduction:

Flavin-binding photoreceptors are critical components across bacteria, plants, animals, and humans, facilitating essential signal transduction pathways and governing vital biological processes such as circadian rhythms and phototropism(1–4). Among flavin-containing photoreceptors, the Light, Oxygen, or Voltage (LOV) sensing flavoproteins are particularly notable for their mechanical response to blue-light stimulation, initiating biochemical and mechanical signaling cascades through structural rearrangements. The ability of LOV proteins to convert absorbed photon energy into controlled mechanical actuation of an associated kinase makes them some of the most attractive protein actuator candidates for bioengineering applications. They have been engineered to control cellular signaling because of their remarkable ability to “turn on” the activity of a tethered protein, simply by shining light. Today, engineered LOV variants are widely used to regulate diverse cellular processes—including cell motility (PA RAC1)(5), nuclear transport (LINuS/LEXY)(6, 7), gene expression (CLASP)(8), ion flux (BLINK)(9), proteolytic cleavage (FLARE)(10), organelle transport (TULIP)(11), prokaryotic transcription (LOVTAP)(12), and developmental signaling (ilid SOS)(13). The *Avena sativa* phototropin 1 LOV2 domain (AsLOV2) exemplifies this capability, utilizing an oxidized flavin mononucleotide (FMN) chromophore non-covalently bound within its structure. Upon illumination with blue light (λ = 450 nm), the flavin mononucleotide (FMN) cofactor transitions to a triplet state and forms a covalent bond with a conserved cysteine residue (C450) that reduces the flavin isoalloxazine ring, triggering conformational changes in AsLOV2 that mechanically actuate a tethered effector protein, thereby optically driving the effector protein function and correlated cellular processes(14–17).

Light activation offers substantial advantages compared to chemical or allosteric modulation, among others, due to its superior temporal and spatial resolution(6, 8, 13). This method enables precise and rapid initiation and termination of biological responses, which are inherently reversible and repeatable without cumulative chemical side-effects or undesirable off-target interactions. However, despite the broad utility and promise of engineered LOV protein variants, current engineering approaches rely on heuristic strategies and directed-evolution screening to achieve or optimize the desired LOV function, due in part to details regarding the molecular basis of converting photon energy into mechanical action remaining elusive(5–8). High-resolution structural studies, including X-ray crystallography of illuminated AsLOV2, do not show any long-range displacement from the dark state equilibrium protein structure(18). Conversely, studies using spectroscopic methods like high-resolution nuclear magnetic resonance (NMR), time-resolved electron paramagnetic resonance (EPR), and Fourier-transform infrared (FTIR) spectroscopy demonstrated that light activation of LOV is accompanied by partial unfolding of the A’α and Jα helix and displacement of the Jα helix from the Iβ-sheet surface upon activation, suggesting a plausible mechanism for transmitting structural change to the kinase domain(19–24).

For LOV domains to function as actuators, mechanical force must arise from coordinated conformational changes that propagate to downstream signaling partners. Yet, the molecular events orchestrating such concerted motions remain unidentified. Water has long been implicated in LOV activation, particularly in the context of the FMN photocycle, where it is proposed to act as an intrinsic base catalyzing redox reaction(25–30). However, the role of water as a mechanical modulator from concerted movement, beyond that of a proton donor/acceptor, has never been considered to our knowledge. Rather than acting solely as a chemical cofactor, water may participate directly in facilitating long-range conformational change that underpin mechanical signaling. A series of studies reported on collective water movement driving protein activation, including tryptophan excitation followed by femtosecond spectroscopy(31–34), enzyme activation by water-mediated thermal network(32), and water network mediated protein dynamical transitions(35), but these are still emerging and highly debated concepts. Direct studies of both protein and water movement upon protein activation are needed to obtain further clarity.

This study tests our hypothesis that the collective action of water hydrating AsLOV2, initiated by a thermodynamic state change, drives the structural transitions of the LOV domain. We investigate changes in the hydration water landscape, concurrent with out-of-equilibrium conformational changes of AsLOV2 initiated by blue-light activation, using advanced spectroscopic and computational methodologies. To monitor light-induced conformational changes, we used time-resolved EPR for real-time protein motion analysis and double electron-electron resonance (DEER) spectroscopy for quantifying intra-protein distance changes. To assess light-induced changes in the hydration environment, we employed ^17^O NMR chemical shift analysis to resolve distinct hydration water populations and Overhauser dynamic nuclear polarization (DNP) to probe protein-adjacent water diffusion dynamics at sub-nanosecond timescales. To evaluate pressure-induced effects, we performed high-pressure DEER spectroscopy to monitor conformational extension, high-resolution solution-state NMR to detect residue-specific structural responses, and atomistic molecular dynamics simulations to model pressure-driven changes in protein conformation and hydration. Together, these measurements reveal a new perspective into concurrent molecular level changes of the protein and water constituents of AsLOV2 upon light and pressure activation.

We identify distinct populations of hydration water at the protein-water interface, including waters tightly bound to the protein surface and lower density, lower entropy, tetrahedrally structured, “wrap” water hydrating small hydrophobic regions of AsLOV2(36–43). Different water populations possess unique solvation thermodynamic and structural properties, as reflected in characteristic three-body hydrogen bond geometries(42). We demonstrate that light- and/or pressure-induced state changes leads to the expulsion of lower entropy wrap water populations that consequently alters the protein’s free energetic landscape, resulting in protein structural rearrangements. Our results support a novel conceptual framework wherein AsLOV2 operates as a hydraulic actuator, with hydration water playing an active role in modulating conformational transitions. This study provides critical insights into the molecular basis of light-driven mechanical actuation mechanism of photosensory domains in the LOV class as used in plant phototropins as well as numerous fungal and bacterial proteins(44–47).

## Results:

### Significant conformational extension in WT and N414Q AsLOV2 upon light activation:

To elucidate the molecular mechanism underlying the light-induced mechanical action of AsLOV2, we examined both the wild-type (WT) protein and the slow-cycling N414Q variant. The N414 residue was thought to be involved in stabilizing FMN through hydrogen bonding and presumed to be compulsory in the activation process of AsLOV2(18, 23, 28, 48, 49). However, the discovery that LOV domains lacking N414 still show strong activity, except with naturally slower photocycle times (Vvd: 18,000 s; YtvA: 3,600 s), gave rise to the hypothesis that water may regulate the N5 proton transfer in FMN, with N414 serving as a catalyst(28, 46, 48). Hence, we selected the N414Q mutant to study in comparison with the WT to examine the role of water in mediating AsLOV2 function.

**Figure 1.**
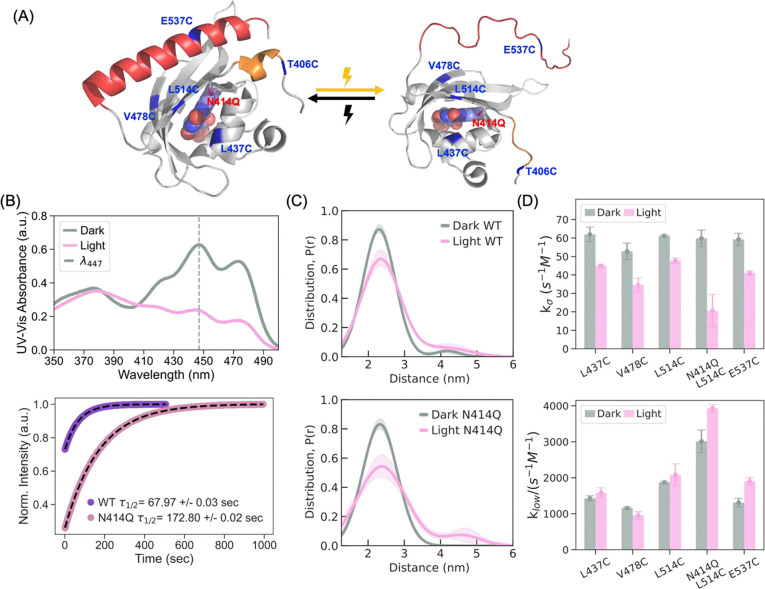
Photoactivation of AsLOV2 induces long range conformational change and modulates interfacial hydration dynamics. (A) Crystal structure of AsLOV2 in the dark state (PDB: 2V1A(62)), highlighting sites labeled with MTSL for ODNP, TiGGER, and DEER measurements (left). Upon photoactivation, the A’α and Jα helices unfold and move further apart, as illustrated on the right. (B) UV-Vis absorbance spectroscopy confirms light-induced activation of AsLOV2. The wild-type protein exhibits a decrease in absorbance at ~450 nm under blue light illumination (top). In the N414Q mutant, time-resolved measurements at 450 nm reveal a 2.5-fold slower return to the dark state, indicating a stabilized lit-state conformation (bottom). (C) The distance distributions obtained with a DeerLab two-Gaussian model (see SI for further details) from the DEER measurements between T406C and E537C show an increase in the population of extended conformations (3–5 nm) in the lit state (top). The N414Q mutant further enriches the extended state population under illumination, consistent with enhanced structural stabilization of the lit conformation (bottom). (D) ODNP measurements reveal that light activation leads to a strong decrease in fast-moving water ($k_{\sigma }$) across all sites, and a smaller increase in slow-moving water ($k_{\text{low}}$) across most sites, suggesting reduced hydration dynamics at the protein interface. This effect is accentuated in the N414Q mutant, particularly at site L514C, where further reduction in $k_{\sigma }$ and increase in $k_{\text{low}}$ indicate enhanced local dehydration and stronger stabilization of the photoactivated state.

Time-resolved UV-Vis measurements confirmed that the FMN dark-state recovery kinetics in the N414Q variant was slowed by a factor of 2.5 (***τ***FMN_N414Q = 172.80 ± 0.02 s) compared to WT AsLOV2 (***τ***FMN_WT = 67.97 ± 0.03 s), while the overall amplitude change in UV-Vis absorption is comparable between the variants, confirming that both variants are fully functional, consistent with the literature(28) (Fig. 1A, B). To investigate the mechanical response of AsLOV2 upon photoactivation in real-time under physiological conditions, we employed time-resolved gadolinium-gadolinium electron paramagnetic resonance (TiGGER) spectroscopy at 240 GHz(21, 50). The results confirmed that both variants exhibit significant and comparable conformational extensions upon blue light illumination, as observed by changes in the dipolar coupling between Gd-TPATCN spin-labels introduced at residues T406C and E537C, located on the N-terminal Aα and C-terminal Jα helices, respectively (SI Fig. S3).

To evaluate the extent of distance changes, we performed DEER(51–54) at cryogenic temperatures on both variants that are spin-labeled with MTSL at T406C and E537C. Samples were photoactivated in solution at room temperature prior to freezing to ensure optimum lit-state population and to preserve the physiologically relevant lit-state structure. DEER of the dark state showed a mean distance at 2.2 nm between the spin labels at sites 406 and 537 in both variants. Upon photoactivation, the amplitude at 2.2 nm dropped with a distance shift toward 3–5 nm to comparable extent in the WT and N414Q variant, indicating light-induced conformational extension in both proteins. (Fig. 1A, C). The observed structural rearrangements in solution state encompassing 1–3 nanometer-scale displacements contrast with X-ray crystal structures that only show sub-angstrom displacements between T406C and E537C. To fully resolve the structural transitions and associated hydration changes upon AsLOV2 activation, we require structural biology tools capable of probing out-of-equilibrium states in solution.

### Reduced surface hydration water dynamics upon light activation:

To examine changes in the property of hydration water upon light activation in solution state, we relied on Overhauser Dynamic Nuclear Polarization (ODNP) that uses nitroxide spin labels (MTSL) at selected AsLOV2 sites to experimentally evaluate the local diffusion dynamics of hydration water associated with AsLOV2. ODNP probes site-specific water dynamics within ~1 nm of the spin label, capturing diffusion processes with correlation times on the order of tens of picoseconds to sub-nanoseconds(55–57). We examined four key sites: site E537C on the Jα helix, which unfolds upon photoexcitation; site L514C on the Iβ sheet, located near the conserved glutamine Q513; site L437C on the Cα helix; and site V478C on the Gβ sheet (Fig. 1A).

ODNP leverages dipolar interaction between the unpaired electron spin (e) of MTSL and the ^1^H nuclei of surrounding water molecules. This interaction results in preferential e-^1^H cross relaxation via the double quantum transition ($w_{2}$) that is facilitated when the instantaneous motion of water near the spin label occurs on a timescale comparable to or faster than the inverse electron spin Larmor precession frequency of 9.8 GHz at 0.35 T. Efficient e-^1^H $w_{2}$ transition followed by physical diffusion of the polarized water from near the spin label to bulk water results in ^1^H NMR signal enhancement of the bulk water signal owing to greater thermal polarization of the electron spin by 658 fold than the ^1^H nuclear spin. By analyzing ODNP enhancements (ε(p)) and ^1^H NMR $T_{1}$ relaxation times, we can extract the coupling factor (ξ) that yields the e-^1^H dipolar correlation times and a local surface water diffusivity, $D_{local}$. Further analysis of the ODNP data allows us to extract two key parameters that report on surface water properties at very different time scales: the cross-relaxivity parameter ($k_{\sigma }$) reflecting on water motion across the dipolar field of the spin label on the picosecond timescale, dictated by the EPR frequency of the spin label (9.8 GHz), and the self-relaxivity parameter ($k_{low}$) capturing bound water population moving on the tens of nanoseconds timescale, primarily influenced by the fluctuation of interactions at the NMR frequency (14.8 MHz)(55–60).

We know from previous studies that the Jα helix unfolds upon light activation(19, 21, 22, 49, 61). The EPR lineshape of the spin label at site E537C on the Jα helix shows motional narrowing in the lit- compared to dark state (SI Fig.S1), suggesting increased side chain dynamics. Based on that, we expected greater water accessibility and greater surface diffusivity ($D_{local}$) (compared to dark state) near site E537C due to increased exposure to freely exchanging water upon unfolding. Spin labels introduced at residues L437C, V478C, and L514C, all of which are on the same face of AsLOV2 as the Jα helix, showed minimal change in the EPR lineshape upon light activation. Surprisingly, across all sites examined, we observed reduced dynamics of water near the protein surface: $D_{local}$ decreased around all sites (SI Fig. S2), accompanied by reduced $k_{\sigma }$ (Fig. 1D top), indicating a slowdown in surface water diffusivity at the protein interface and a slightly increased $k_{low}$ (Fig. 1D bottom) indicating a greater population of slow moving, bound, water population upon light activation. Similar trends were observed in the slow-cycling N414Q mutant at site L514C, with more pronounced changes observed in its hydration environment compared to WT upon light activation (Fig. 1D). For hydration water dynamics to slow down at sites that are not experiencing restriction in movement or accessibility to water or even experiencing an increased protein side chain mobility such as site E537C, the composition of hydration water around this site must be altered towards an increased fraction of hydration water population with slower mobility upon light activation.

We hypothesize that at least two distinct water populations besides bulk water make up the hydration layer of AsLOV2, (i) strongly hydrogen-bonded (bound) water with slower dynamics and (ii) hydration water with greater tetrahedral structuring and lower entropy wrapping over small hydrophobic protein sites (wrap water)—a categorization consistent with recent studies reporting on distinct water types with different structural properties, high-density, icosahedral and low-density, tetrahedral water, coexisting in aqueous solutions with varying populations(36–43). The apparent slowing of surface water dynamics can be due to an increased population of the slower diffusing, bound water and/or the eviction of the faster diffusing, wrap water into bulk water. We note that both hydration water populations have slower dynamics than bulk water. The latter scenario, the selective expulsion of wrap to bulk water, would be thermodynamically favorable due to overall entropy increase. If light activation is accompanied by selective expulsion of wrap water, the resulting increase in entropy upon dehydration would thermodynamically favor and stabilize the lit state. The N414Q variant shows greater degree of dehydration according to ODNP data, suggesting that the N414Q mutation further stabilizes the lit state, in agreement with prior reports(23, 28, 49). This is consistent with the N414Q mutation showing uncompromised photoactivity and conformational extension according to UV-Vis, TIGGER and DEER, just with slower lit-to-dark state recovery kinetics. We next seek direct experimental evidence using ^17^O NMR chemical shift analysis to support this claim.

### ^17^O NMR chemical shift shows distinct hydration water populations:

The ^17^O NMR chemical shift of freely tumbling water is exquisitely sensitive to the electron density on the oxygen atom. It can detect subtle changes such as 0.01 Å differences in O-H covalent or hydrogen bond lengths and whether three or four hydrogens are bound to the central oxygen. In solution state under free tumbling conditions, the ^17^O NMR shift should not be altered significantly by the second-order quadrupolar effect. Hence it offers a direct experimental signature for different water structures(63–69). These dynamic structures are persistent in solution state as recently demonstrated for lipid membrane surfaces by Zhang et al.(70) Furthermore, a series of studies of water/glycerol and water/DMSO mixtures identified bound and wrap water, categorized by differences in their tetrahedral content and reflected in the three body angles around 109°, to be the two dominant liquid water structural archetypes(39, 42). These water structures have distinct hydrogen bond lengths, and hence different ^17^O chemical shifts, with wrap water exhibiting a downfield shift relative to bound water(71). Bulk or hydration water contain both populations in varying proportions that are also in dynamic exchange. The question is whether and how their makeup in water associated with AsLOV2 changes upon light activation. To answer this question experimentally, we increased the ^17^O isotope concentration by adding 40% ^17^O enriched water to a buffered AsLOV2 solution and measured ^17^O NMR spectra of WT and slow-cycling N414Q in solution state at 18.8 T and 294 K under MAS (10 kHz). The ^17^O $T_{1}$ relaxation time of the solution containing WT AsLOV2 is 4.07 ms and N414Q is 5.15 ms. Both values are significantly shorter than the 7.05 ms value of pure water doped with the same ^17^O content, suggesting that hydration water populations with distinctly slowed dynamics are present in AsLOV2 solutions and measurable by ^17^O NMR.

**Figure 2.**
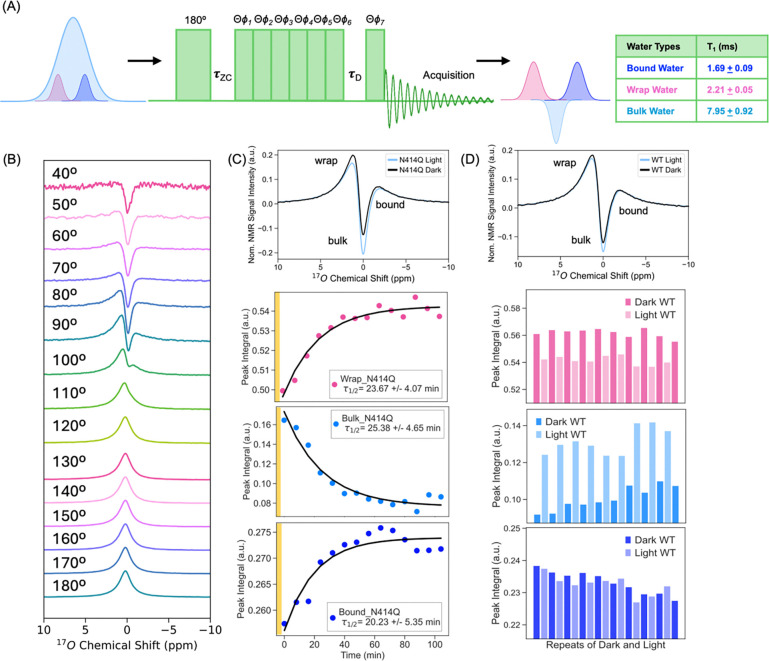
Light-Driven Redistribution of Distinct Water Populations in AsLOV2 Revealed by High-Field ^17^O MAS NMR. (A) Single-pulse 1D 17O MAS NMR spectrum of 40% 17O-enriched buffered AsLOV2 solution, acquired with bulk water suppression at 800 MHz and a spinning rate of 10 kHz ± 3 Hz at room temperature, reveals three spectrally distinct water populations. Bulk water exhibits the longest $T_{1}\;(7.95\pm 0.92\;ms)$, bound water shows the shortest $T_{1}\;(1.69\pm 0.09\;ms)$ and wrap water with intermediate $T_{1}\;(2.21\pm 0.05\;ms)$. (B) Experiments conducted with fixed $\tau_{ZC}=2.7\;ms$ and $\tau_{D}=0$, while varying the flip angle θ, show that two water populations—assigned as “bound” and “wrap” water—are preferentially observed at low flip angles (<100°). (C) Due to its slower dark-state recovery, the N414Q mutant allows time-resolved tracking of hydration changes. Upon return to the dark state, the system exhibits gradual: recovery of wrap water, reduction in bulk water signal, and recovery of bound water, all occurring on a timescale of $\tau_{1/2}∼20\;minutes$, consistent with photocycle kinetics measured by time-resolved UV-Vis spectroscopy at 277 K. (D) In the wild-type protein, light activation leads to rapid and reversible: expulsion of wrap water, an increase in bulk water signal, and minimal change in bound water levels, as observed across repeated photocycles.

We used a pulse sequence established by Zhang et al (70) to suppress the ^17^O NMR signal from bulk water at the zero crossing of bulk water at $\tau_{zc}$ 2.70 ms delay after an inversion pulse (Fig. 2A), so that the ^17^O NMR signal of the hydration water would be enhanced relative to bulk water if their $T_{1}$ is distinct from that of bulk water. With $\tau_{zc}$ set to 2.70 ms and θ at 90°, the water-suppressed ^17^O NMR spectrum showed distinct signatures (Fig. 2C top, 2D top) up- and down-field of the bulk water peak. When varying the flip angle (θ) from 40° to 180° at fixed τzc=2.7ms and $\tau_{D}=0$, we observed more effective suppression of bulk water signal at lower flip angles (θ < 100° for WT and θ < 130° for N414Q), with more clearly resolved features corresponding to hydration water consistent with the observations by Zhang et al. As a control, we measured a 40% ^17^O enriched pure water sample under identical conditions, where no such distinct features were observed across the 0° to 180° range, confirming that the spectral signatures detected in the AsLOV2 samples arise from hydration water associated with the protein.

The greater spectral resolution at lower θ values was used to measure spectrally resolved $T_{1}$ relaxation times (SI Fig. S4B) that reveal three discrete value ranges centered around: 2.21 ± 0.05 ms, 7.95 ± 0.92 ms and 1.69 ± 0.09 ms at ^17^O NMR shift ranges 8.0 to 0.5 ppm, 0.4 to −0.4 ppm and −0.5 to −8.0 ppm, respectively, relative to bulk water at 0 ppm. The spectral population exhibiting the longest $T_{1}$ time, implying faster water dynamics, matched the $T_{1}$ time of pure water of 7.05 ms, and was therefore assigned to bulk water corresponding to the 0.4 to −0.4 ppm range. The observation that distinctly shorter $T_{1}$ values are found for the down- and up-field shifted spectral region suggests these are protein-associated hydration water populations. Of these, we assign the down-field shifted signature as wrap water (2.21 ± 0.05 ms), consistent with DFT predictions for wrap water(71). The origin of the up-field shifted peak is less clear, but its shorter $T_{1}$ (1.69 ± 0.09 ms) implies slower dynamics, and therefore we assign it to bound water populations. Since these populations of water—bulk, bound, and wrap—are in dynamic exchange with each other in a dilute solution as our system, we anticipate the observed lineshape to be a convolution of spectral signatures of multiple populations that cannot be readily distinguished. Still, there are three clearly distinct spectral regions identified by different ^17^O NMR $T_{1}$ values; hence we will focus on amplitude changes in each of these spectral regions in the light and dark states.

With spectroscopic signatures of wrap, bound and bulk water as experimental handles, we next examine changes in their amplitudes upon activation of AsLOV2 with light. These measurements were conducted at a lower temperature of 277 K to slow the exchange rate between hydration water and bulk water, thereby enhancing the resolution and sensitivity for detecting light-induced changes in the hydration water composition. Light activation induces a reversible decrease in wrap water and a concurrent increase in bulk water, with a minimal to the bound water population in WT (Fig. 2D). The fast recovery time of $\tau_{\text{FMn\_WT}\;}∼3.5\;min$ (277 K) of WT did not readily allow for time-resolved measurements, given the acquisition time of 3 min for a ^17^O NMR spectrum. The slower dark state recovery time of N414Q of $\tau_{\text{FMN\_N414Q}\;}∼23\;min$ allowed us to readily capture the recovery of wrap water and loss of bulk water population upon light activation as a function of time, yielding a time constant of ~20 min for all three populations, which is on par with the recovery time according to UV-Vis absorption at 277 K (Fig. 2C, S5). Time resolved measurements reveal that the bound water population also changes, but to a much smaller extent than changes in the wrap and bulk water populations. Hence, changes in the ^17^O NMR chemical shift in solution state capture subtle changes in bound water populations, while more prominently reflecting the light-induced expulsion of wrap water into bulk, with reversible recovery upon return to the dark state. Combined with ODNP results showing that protein-adjacent hydration water dynamics is slowed down by light activation, we can conclude that the light activated state is predominantly hydrated by slow diffusing, bound water upon eviction of the more dynamic, wrap water, in an entropy-driven process.

Our results reveal that a collective eviction of wrap water ensues light activation of AsLOV2, given that ^17^O NMR of water captures the change of the entire population of wrap water population without site specificity. This raises a compelling question: could the movement of hydration water be a key modulator of the mechanical actuation of AsLOV2?

### Pressure to replace light as an inducer of AsLOV2 activation:

If water movement induces conformational changes that accompany AsLOV2 activation, then any perturbation that induces a concerted eviction of structured hydration water could serve as a trigger. Naturally, this suggests pressure can replace light as an activator. We tested this hypothesis using a combination of computational and experimental methods. Computationally, we perform molecular dynamics (MD) simulations of AsLOV2 at 3 kbar and compare to 1 bar to determine whether elevated pressure induces conformational changes consistent with light activation of AsLOV2 and eviction of wrap water. Unlike pressure activation, light activation involves changes in the electronic structure of FMN and surrounding molecules, and hence is outside the reach of classical MD. Experimentally, we performed DEER to measure distance change across spin labeled sites T406C-E537C and 3D HNCO NMR to evaluate changes in the amide ^1^H-^15^N and carbonyl ^13^C chemical shifts of AsLOV2 residues under high pressure and compared these to changes upon light activation.

Proteins in solution exist as an ensemble of conformational substates, each with distinct partial molar volumes. High pressure perturbs these equilibria by stabilizing conformations with reduced volume, often shifting populations toward folding intermediates or excited states that are otherwise sparsely populated under ambient conditions. While this concept of populating sparsely populated, excited states by high-pressure NMR(72–76) or EPR(77–81) has been established, the factors that govern intrinsic protein volume change and compressibility are only partially understood. We hypothesize that different populations of wrap and bound water constituting the hydration shell plays a critical role in pressure-induced conformational transitions. We propose that the lower density wrap water has greater compressibility and is preferentially displaced upon pressure increase, leaving behind the higher density hydration layer in which water remains more strongly bound to the protein surface. This transition may stabilize the protein’s excited state by increasing the total entropic contributions, minimizing the overall protein volume, and promoting solvation of newly exposed residues.

### Molecular dynamics simulations show expulsion of wrap water in pressure-activated AsLOV2:

**Figure 3.**
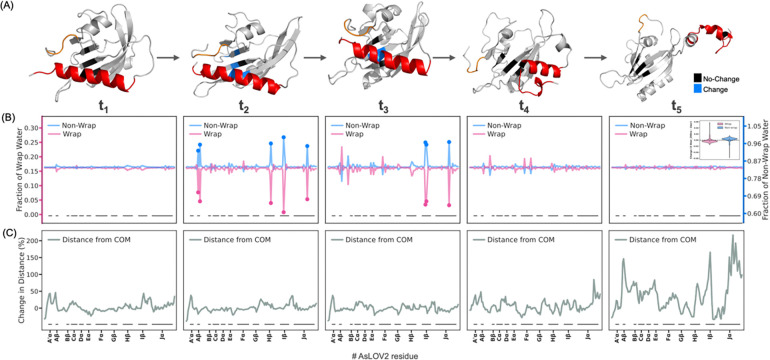
High-pressure molecular dynamics simulations reveal site-specific redistribution of water associated with pressure-induced unfolding in AsLOV2. (A) High-pressure molecular dynamics simulations reveal pressure-driven partial unfolding of AsLOV2, initiated by unraveling of the A’α helix and extension of the Jα helix, mirroring the structural changes observed upon photoactivation. (B) Snapshots from the 3 kbar unfolding trajectory reveal that wrap water remains stably associated with compact regions of the protein, such as at t_1_ (5% of total simulation time). A notable decrease in tetrahedral wrap water is observed by t_2_ (12% of total simulation time), particularly around the Aβ, Hβ, Iβ, and Jα regions, and becomes more pronounced near Iβ and Jα by t_3_ (27% of total simulation time). Jα begins unfolding at t_4_ (53% of total simulation time) and fully undocks by t_5_ (100% of total simulation time), and the wrap water redistributes to resemble the 1 bar state, though with slightly reduced overall occupancy (inset). Pink dots indicate residues with >50% reduction in wrap water relative to the mean across all residues. while blue dots represent non-wrap water (total hydration shell water minus wrap water), which increases at corresponding residues showing wrap water loss at t_2_ ns and t_3_ ns. (C) Time-resolved residue-wise structural deviations relative to the 1 bar state reveal that pronounced changes begin to occur around t_5_ ns, particularly in the Iβ and Jα regions, suggesting that hydration loss precedes major conformational transitions.

To test this hypothesis, we performed MD simulations of AsLOV2 at room temperature (298 K) under both ambient (1 bar) and elevated (3 kbar) pressure by computing transfer free energies(82) in implicit solvent for 200 ns following the protocol outlined by Arsiccio and Shea(82). Our results reveal a pressure-driven conformational transition consistent with light-induced activation, in which partial unfolding of AsLOV2 is initiated by the unraveling of the A’α helix, followed by extension and unfolding of the Jα helix. These helices move opposite to one another, with the Jα helix becoming more extended over longer equilibration times at high pressure (Fig. 3A).

To determine whether pressure-induced unfolding involves the expulsion of tetrahedral like wrap water, we analyzed the water structure by considering the three-body-angle (3BA) formed by a central oxygen and two neighboring oxygens from hydrogen-bonded water molecules(83, 84) using explicit-solvent simulations with the CHARMM36m force field and TIP3P-CHARMM water model(85). 196 representative unfolded conformations were obtained via RMSD clustering of the last 100ns of 200 ns long unfolding trajectories, following the method of Daura et al.(86) implemented in GROMACS(87), and change-point detection of Solvent Accessible Surface Area (SASAR), implemented using the Python ruptures package(88). Each unfolded conformation was then inserted into a new simulation box alongside explicit water and simulated for 500 ns. We examined the distribution of water 3BA within the protein hydration shell and quantified the fraction of low-density tetrahedral water (3BA near 109.5°) at both 1 bar and 3 kbar-unfolded states for the 200–500 ns simulation window. We find a subtle yet consistent decrease in the population of the tetrahedral wrap water across most residues, including the A’α and Jα helices, accompanied by an increase in non-tetrahedral water populations (three-body angle 40°-100° or 120°-180°) with rising pressure (SI Fig. S11).

This observation suggests that pressure-induced unfolding of AsLOV2 is closely associated with the eviction of tetrahedral wrap water into bulk water, consistent with experimental observation. This mechanism underscores that compressibility of a protein is a function of the protein-associated water structural properties and populations. To date, protein compressibility has been conceptualized in terms of protein-driven determinants such as packing defects and cavities, while our findings demonstrate that modulating the population of waters of different density is a major factor. In fact, cavity- or solvation-based models for compressibility may be strongly correlated. Future studies will have to investigate whether wrap water eviction is a general mechanism in high pressure-mediated protein conformational changes.

### DEER studies show that light and pressure are additive factors for AsLOV2 activation:

**Figure 4.**
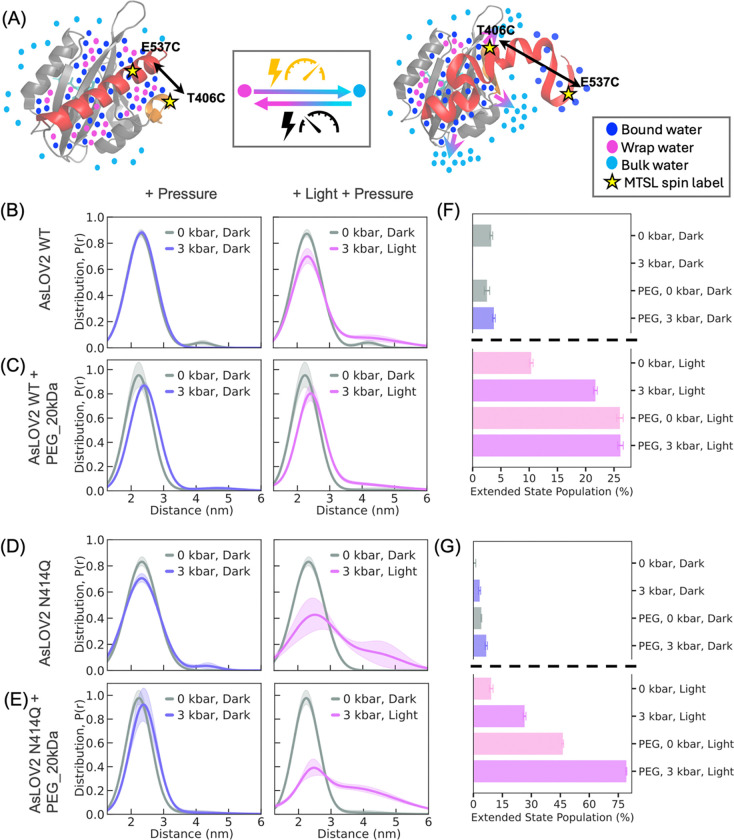
DEER measurements show that pressure and light co-operatively enhance the population of extended state conformations in AsLOV2, with effects amplified by molecular crowding (see SI for details of the analysis of the DEER data). (A) Schematic of MTSL spin-labeled AsLOV2 at positions T406C and E537C, illustrating conformational extension and concurrent release of wrap water upon stimulation by light and/or pressure. (B) Left: DEER-derived distance distribution (𝑟) shows that application of 3 kbar pressure alone did not result in a detectable structural extension from the dark-state equilibrium conformation in the WT. Right: Combined light and pressure further increase the extended population to ~21%, highlighting an additive effect. (C) In the presence of PEG_20kDa as a molecular crowder, pressure alone increases the WT extended population to ~4% (left), and light plus pressure drives it further to ~26% (right), suggesting enhanced conformational responsiveness due to internal osmotic pressure. (D) In the N414Q mutant (no crowder), pressure alone results in ~3% of the population in the extended conformation (left), while light plus pressure increases this to ~26% (right), indicating greater stabilization of the lit state. (E) In the presence of PEG_20kDa, the N414Q mutant shows even stronger pressure sensitivity: ~7% in the extended state under pressure alone (left), and ~79% with combined light and pressure (right). (F–G) Summary of population shifts under different conditions, indicating that both external (hydrostatic) and internal (osmotic) pressure cooperatively enhance conformational extension in AsLOV2, with the N414Q mutant exhibiting a greater propensity for adopting the excited-state conformation.

Next, we experimentally compare effects of both light and pressure (3 kbar)(77–81) on the conformational changes in AsLOV2 using DEER of doubly spin labeled WT and N414Q AsLOV2 at sites T406C and E537C. DEER measurements in the dark state revealed a stable inter-residue distance of 2.2 nm in both variants, while light activation results in a comparable extension in WT and N414Q by 1–3 nm (Fig. 1A, C). Application of 3 kbar pressure alone (see SI for method) did not result in a measurable shift from this equilibrium distance in the WT (Fig. 4B, F). In contrast, light activation alone resulted in ~10% of the WT population adopting an extended conformation (Fig. 1A, C). Interestingly, when both stimuli, light and pressure, were applied simultaneously, the effects were additive, with 21% of the population adopting the extended conformation, surpassing the effect of either stimulus alone (Fig. 4B, F).To examine the effects of higher pressure, given that our experimental setup was limited to 3 kbar, we increased the internal osmotic pressure using a molecular crowder (PEG 20 kDa). PEG alone induced a modest shift, with ~3% of the population adopting an extended conformation at an average P(r)~4nm. When PEG was combined with 3 kbar pressure, the population in the extended state of WT increased to 4%, suggesting a marginal additive effect. Finally, the combination of light, pressure, and PEG yielded the largest conformational shift, with 26% of the WT population adopting the extended conformation (Fig. 4C, F).

We next examine the impact of pressure on the slow-cycling N414Q variant that inherently stabilizes the excited state more effectively than the WT. Interestingly, light activation alone resulted in 9% of the N414Q population in the extended state, comparable to the 10% observed in WT. However, a greater increase in the excited-state population compared to the WT is seen under the effect of 3 kbar pressure alone with 3% in the extended population (as opposed to 0) and 26% when both triggers were combined (Fig. 4D, G). The application of PEG in addition to pressure and light yielded the highest observed population of the extended conformation of 79% (Fig. 4E, G). In light of our ODNP results suggesting that N414Q undergoes greater extent of dehydration, it is notable that pressure activation has a greater effect on N414Q compared to WT.

These results provide experimental evidence that pressure induces conformational extension in AsLOV2 that closely parallels the effect by light, further strengthened by the observation that the effect of pressure and light are additive. The findings that a molecular crowder such as PEG can also promote the activation of AsLOV2 supports our hypothesis that, whether induced by external pressure, osmotic stress, or light, hydraulic action is at the core of AsLOV2 activation. This study demonstrates that nature might be leveraging both external and internal pressure to regulate functional conformational transitions in LOV domains, rendering them mechanosensitive.

### HNCO NMR shows pressure activation induces similar conformational changes as blue light:

The MD and DEER results offered motivation to further extend this investigation to experimentally capture pressure-induced structural changes at residue-level resolution across the entire protein. To accomplish this, we employed high-pressure three-dimensional HNCO (^1^H/^15^N/^13^C) NMR spectroscopy on AsLOV2, across a pressure range up to 2.5 kbar using a high-pressure ceramic tube (Daedalus Innovations). This approach enabled site-specific quantification of pressure-induced chemical shift perturbations in ^1^H, ^15^N, and ^13^C nuclei of the protein backbone.

**Figure 5:**
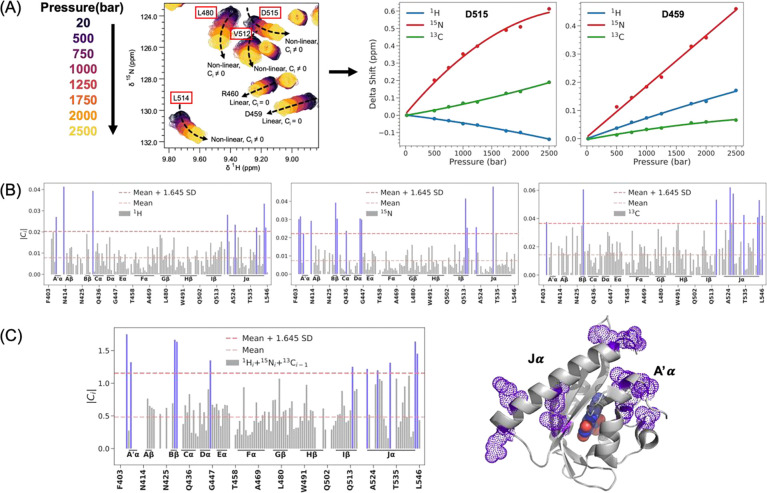
Residue-resolved analysis of pressure-induced conformational changes in AsLOV2 using high-pressure 3D HNCO NMR. (A) Representative two-dimensional ^15^N/^1^H projections from high-pressure HNCO spectra collected across a pressure range up to 2.5 kbar. Peak trajectories illustrate pressure-dependent chemical shift changes, including both linear and nonlinear behaviors across different residues. (B) Quadratic fitting of chemical shift changes for three backbone nuclei—^1^H, ^15^N, and ^13^C—was used to extract nonlinear coefficients ($c_{i}$) for each residue. Bar plots show the distribution of absolute nonlinear coefficients $\left|c_{i}\right|$ across the protein for each nucleus, with horizontal lines indicating the statistical cutoffs (mean, and mean + 1.645 SD). (C) A composite nonlinearity score was calculated by normalizing and summing the nonlinear coefficients across all three nuclei (^1^H_*i*_ + ^15^N_*i*_ + ^13^C_*i-1*_), yielding an integrated metric for pressure sensitivity per peptide group. Structural mapping of the top 10% of residues with the largest deviations from the mean onto the AsLOV2 crystal structure (PDB: 2V1A (62)) reveals that the most pressure-sensitive regions are localized to the A′α and Jα helices and their adjacent segments. These helices are also known to be functionally critical in the light activation mechanism of AsLOV2, suggesting a shared structural pathway for both light- and pressure-induced activation via hydration-mediated “hydraulic” coupling.

Representative two-dimensional ^15^N/^1^H projections from the 3D NMR dataset illustrate individual resonances undergoing either linear or nonlinear chemical shift changes with increasing pressure (Figure 5A). To systematically quantify these shifts across all three nuclei, we performed least-squares fitting of pressure-dependent chemical shifts for each residue to a quadratic function of pressure:

$$\delta_{i}\left(ppm\right)=a_{i}+b_{i}p+c_{i}p^{2},$$

where $\delta_{i}$ is the chemical shift (ppm) for the i-th residue at pressure p (bars), $a_{i}$ is the chemical shift at 20 bar, and $b_{i}$ and $c_{i}$ are the linear and nonlinear coefficients, respectively. This empirical model is analogous to the thermodynamic expansion of Gibbs free energy as a function of pressure, allowing for a biophysical interpretation of the coefficients: $b_{i}$ corresponds to changes in the partial molar volume, while $c_{i}$ captures changes in compressibility of site i.

Residues exhibiting linear pressure shifts ($c_{i}=0$) are interpreted as undergoing population redistributions among conformational substates within the native ensemble, usually due to isotropic compression of the protein by the applied pressure. In contrast, residues with significant nonlinear components ($c_{i}\neq 0$) suggest a population shifts toward a pressure-favored conformational ensemble characterized by altered compressibility that correspond to out-of-equilibrium states under ambient pressure(72–76). To identify residues undergoing the most pronounced conformational changes, we calculated the absolute value of the nonlinear coefficient ($c_{i}$) for each residue and applied statistical thresholds based on mean and standard deviation (mean + 1.645 SD). This threshold captures the top 10% of residues with the largest deviations from the mean, designating them as major sites of significant pressure-induced change. (Figure 5B).

The chemical shift behavior of individual nuclei offers distinct structural insights: ^1^H shifts correlate strongly with changes in the H···O hydrogen bond distance in NH···O=C motifs; whereas the ^15^N shifts are sensitive to variations in backbone torsion angles (72–76). In contrast, ^13^CO chemical shifts, which have been less commonly studied experimentally, report on both parameters but offer a more global structural readout of changes in the secondary structure. To integrate the complementary information provided by each nucleus and obtain a comprehensive residue-level measure of nonlinear response, we normalized the nonlinear coefficients across the three nuclei and calculated a combined nonlinearity score for each peptide group by summing the normalized values. This integrated metric provided a holistic, residue-level assessment of pressure-sensitive conformational changes throughout the protein backbone (Figure 5C).

The top 10% of residues with the largest deviations from the mean were concentrated primarily within A′α (T406, L408), Bβ (F429, A430), Dα (L446), Iβ, (L514), Jα (R521, R526, I532, K544, E545). These findings show that the N- and C-terminal regions of AsLOV2 undergo substantial pressure-induced conformational changes. Notably, these regions correspond precisely to critical structural elements involved in the blue-light activation mechanism(22). This concordance implies that high pressure does not indiscriminately destabilize the protein structure but activates AsLOV2 by populating excited structural states, similar to light-induced signaling, consistent with the DEER results. We propose a common hydration-mediated “hydraulic” coupling mechanism underlying both pressure- and light-mediated activation. This conclusion is further reinforced by our DEER results, which independently revealed related conformational changes that drive the A’α and Jα helices apart by several nanometers, implying that the Jα helix undocks from the Iβ surface, induced by additive effects of pressure and light activation.

### Does hydraulic action follow or precede protein activation?

The final question that we ask ourselves is whether the concerted water action precedes or follows conformational changes. The observation that pressure can engage in comparable and additive activation as light, and that low-density wrap water populations are selectively evicted upon light activation (according to ODNP and ^17^O NMR) and pressure activation (according to MD), suggests that light activation gives rise to hydraulic action that drives protein activation. However, with the current technology available, direct access to the sequence of events of protein and water movement of AsLOV2 under physiological condition is only possible computationally, informing our final quest whether pressure-induced eviction of wrap water precedes the structural transformation of AsLOV2.

To investigate the temporal relationship between pressure-induced conformational changes and hydration dynamics, we analyzed multiple time points along the high-pressure (3 kbar) MD trajectory. The A’α helix undergoes complete unfolding within the first 2.5% of total simulation time (SI Fig.S12), yet the overall distribution of wrap water remains largely unchanged across residues up to t_1_ (5% of total simulation time). At around t_2_ (12% of total simulation time) we observe a sudden and significant decrease in tetrahedral wrap water surrounding residues in the Aβ (V416, T418), Hβ (L496), Iβ (I510), and Jα (A536) regions, while the Jα helix and Hβ and Iβ fold remain fully folded. By t_3_ (27% of total simulation time), further eviction of wrap water is observed, but more pronounced around the Iβ (I510, G 511) and Jα (A536) domains, while the Jα helix is still structurally fully intact. At t_4_ (53% of total simulation time), no further significant changes in local hydration are detected, while first signs of Jα helix unfolding emerge at A536. Finally, by t_5_ (100% of total simulation time), a complete redistribution of wrap water that resembles the hydration pattern at 1 bar is observed, albeit with a slightly reduced overall population of wrap water, while the Jα helix has fully undocked and moved away from the A’α helix, (Fig. 3B). This analysis shows that eviction of wrap water occurs between t_1_ and t_3_. To assess the magnitude of global structural change at each time point, we computed residue-wise displacement relative to the center of gravity of the initial 1 bar structure. Minimal fluctuations were observed across the protein up to t_4_, consistent with the stability of key secondary structure elements observed. Around t_5_, pronounced global structural deviations emerge, particularly in the Iβ and Jα regions, confirming that changes in hydration precede major conformational transitions in the protein (Fig. 3C).

## Discussion and Conclusion:

These results support a model in which pressure-induced unfolding is driven by the concerted loss of structured hydration water, particularly near the critical Jα helix. What began as an exploration to capture light-driven conformational extension of AsLOV2 evolved into the recognition of a novel mode of protein activation—one associated with a concerted and reversible motion of water in and out of the protein. Using a combination of time-resolved EPR and DEER spectroscopy, we observed a long-range structural extension upon blue-light illumination of the tip of the Jα helix relative to A’α, a hallmark of functional LOV activation. ODNP experiments revealed a surprising slowdown in surface water diffusivity upon light activation, even at highly water-accessible protein surface sites, hinting at a light-triggered restructuring of the hydration shell of AsLOV2. High field ^17^O NMR allowed us to spectroscopically resolve the distinct populations of bulk, wrap, and bound water, and crucially, track their collective and reversible redistribution during photoactivation, and validating our hypothesis that wrap water is selectively evicted, transforming into bulk water, and recalled to hydrate AsLOV2 in the dark state. Molecular dynamics simulations under elevated pressure not only recapitulated the light-induced conformational transition but revealed pressure as an alternative activation inducer, acting through the same mode of expulsion of low-density, tetrahedral wrap water. These predictions were experimentally confirmed by high-pressure DEER, which showed pressure-induced extension, further amplified under conditions of molecular crowding. High-pressure 3D HNCO NMR uncovered residue-specific nonlinear responses concentrated in helices known to mediate light signaling, reinforcing the connection between hydration modulation and structural activation. Lastly, MD simulations revealed that concerted water eviction precedes protein conformational extension, offering further support for the thesis of hydraulic activation of AsLOV2.

Together, our results present a novel conceptual framework of AsLOV2 as a hydraulic actuator. Unlike earlier models that positioned water as a passive solvent or as molecular constituents serving as a proton donor or acceptor, we show that water—specifically the structured populations at the protein interface—can serve as a hydraulic fluid to exert mechanical work in a thermodynamic process. We offer a testable hypothesis: hydraulic activation is a generalized mechanism of mechanosensitive LOV domains that produces mechanical work upon light activation. This concept provides a new lens for understanding protein responsiveness to both optical and mechanical cues. The discovery that pressure, like light, can drive this transition has implications for designing externally controllable protein-based systems. This is supported by demonstrations of protein-based switches existing in pre-existing equilibria that are modulated by specific stimuli, e.g. light with AsLOV2 (89), but also by point mutations (90) and general thermodynamic variables such as temperature (91). Our work extends this to an often-underused parameter – pressure – and offers a new mechanistic framework of hydraulic action underpinning protein activation. This study advances the blueprint for rationally engineering and testing optogenetic tools and actuators whose activity can be finely tuned for applications in synthetic biology, smart materials, and targeted therapeutic modulation.

While our study provides converging evidence for a hydration-mediated activation mechanism in AsLOV2, several opportunities remain to sharpen and expand this framework. Time-resolved methods such as pressure-jump NMR, EPR and diffraction-based methods will help resolve the temporal sequence of hydration change and conformational transition events. Additionally, whether the eviction of wrap water alone, independent of chromophore chemistry, is sufficient to promote activation remains to be explored through the study of other LOV protein variants. Future studies could examine whether modulating solvent characteristics near ambient pressure biases the protein toward activation-prone conformations in the absence of light or mechanical triggers. The combination of electronic structure calculation and atomistically detailed MD simulations may offer more direct insight into light-activated changes in protein-associated water and offer more quantitative insight into the hydrogen-bonding geometries and electronic environments that distinguish wrap, bound, and bulk water populations. Furthermore, the development of site-specific ^17^O NMR methods would enable spatially resolved mapping of hydration dynamics near key structural regions of immobilized AsLOV2 in a near solid state. Extending the here presented studies to other LOV proteins will be critical to determine whether hydraulic activation is a broadly conserved strategy or a finely tuned adaptation, offering deeper insight into how proteins use water not just as solvent, but as an active medium for transducing environmental signals into mechanical motion.

## Supplementary Material

Supplement 1

## References

[1] Conrad K. S., Manahan C. C., Crane B. R., Photochemistry of flavoprotein light sensors. Nat Chem Biol 10, 801–809 (2014). 25229449 10.1038/nchembio.1633 PMC4258882

[2] Jentzsch K., Wirtz A., Circolone F., Drepper T., Losi A., Gärtner W., Jaeger K.-E., Krauss U., Mutual Exchange of Kinetic Properties by Extended Mutagenesis in Two Short LOV Domain Proteins from Pseudomonas putida. Biochemistry 48, 10321–10333 (2009). 19772355 10.1021/bi901115z

[3] Kasahara M., Swartz T. E., Olney M. A., Onodera A., Mochizuki N., Fukuzawa H., Asamizu E., Tabata S., Kanegae H., Takano M., Christie J. M., Nagatani A., Briggs W. R., Photochemical Properties of the Flavin Mononucleotide-Binding Domains of the Phototropins from Arabidopsis, Rice, andChlamydomonas reinhardtii. Plant Physiology 129, 762–773 (2002). 12068117 10.1104/pp.002410 PMC161699

[4] Chaves I., Pokorny R., Byrdin M., Hoang N., Ritz T., Brettel K., Essen L.-O., van der Horst G. T. J., Batschauer A., Ahmad M., The Cryptochromes: Blue Light Photoreceptors in Plants and Animals. Annual Review of Plant Biology 62, 335–364 (2011). 10.1146/annurev-arplant-042110-103759 21526969

[5] Wu Y. I., Frey D., Lungu O. I., Jaehrig A., Schlichting I., Kuhlman B., Hahn K. M., A genetically encoded photoactivatable Rac controls the motility of living cells. Nature 461, 104–108 (2009). 19693014 10.1038/nature08241 PMC2766670

[6] Niopek D., Benzinger D., Roensch J., Draebing T., Wehler P., Eils R., Di Ventura B., Engineering light-inducible nuclear localization signals for precise spatiotemporal control of protein dynamics in living cells. Nat Commun 5, 4404 (2014). 25019686 10.1038/ncomms5404 PMC4104460

[7] Niopek D., Wehler P., Roensch J., Eils R., Di Ventura B., Optogenetic control of nuclear protein export. Nat Commun 7, 10624 (2016). 26853913 10.1038/ncomms10624 PMC4748110

[8] Chen S. Y., Osimiri L. C., Chevalier M., Bugaj L. J., Nguyen T. H., Greenstein R. A., Ng A. H., Stewart-Ornstein J., Neves L. T., El-Samad H., Optogenetic Control Reveals Differential Promoter Interpretation of Transcription Factor Nuclear Translocation Dynamics. Cell Systems 11, 336–353.e24 (2020). 32898473 10.1016/j.cels.2020.08.009 PMC7648432

[9] Papanatsiou M., Petersen J., Henderson L., Wang Y., Christie J. M., Blatt M. R., Optogenetic manipulation of stomatal kinetics improves carbon assimilation, water use, and growth. Science 363, 1456–1459 (2019). 30923223 10.1126/science.aaw0046

[10] Wang W., Wildes C. P., Pattarabanjird T., Sanchez M. I., Glober G. F., Matthews G. A., Tye K. M., Ting A. Y., A light- and calcium-gated transcription factor for imaging and manipulating activated neurons. Nat Biotechnol 35, 864–871 (2017). 28650461 10.1038/nbt.3909 PMC5595644

[11] Strickland D., Lin Y., Wagner E., Hope C. M., Zayner J., Antoniou C., Sosnick T. R., Weiss E. L., Glotzer M., TULIPs: tunable, light-controlled interacting protein tags for cell biology. Nat Methods 9, 379–384 (2012). 22388287 10.1038/nmeth.1904 PMC3444151

[12] Strickland D., Moffat K., Sosnick T. R., Light-activated DNA binding in a designed allosteric protein. Proceedings of the National Academy of Sciences 105, 10709–10714 (2008). 10.1073/pnas.0709610105 PMC2504796 18667691

[13] Johnson H. E., Goyal Y., Pannucci N. L., Schüpbach T., Shvartsman S. Y., Toettcher J. E., The Spatiotemporal Limits of Developmental Erk Signaling. Developmental Cell 40, 185–192 (2017). 28118601 10.1016/j.devcel.2016.12.002 PMC5289754

[14] Swartz T. E., Wenzel P. J., Corchnoy S. B., Briggs W. R., Bogomolni R. A., Vibration Spectroscopy Reveals Light-Induced Chromophore and Protein Structural Changes in the LOV2 Domain of the Plant Blue-Light Receptor Phototropin 1. Biochemistry 41, 7183–7189 (2002). 12044148 10.1021/bi025861u

[15] Petersen J., Inoue S., Kelly S. M., Sullivan S., Kinoshita T., Christie J. M., Functional characterization of a constitutively active kinase variant of *Arabidopsis* phototropin 1. Journal of Biological Chemistry 292, 13843–13852 (2017). 28663371 10.1074/jbc.M117.799643 PMC5566536

[16] Schleicher E., Kowalczyk R. M., Kay C. W. M., Hegemann P., Bacher A., Fischer M., Bittl R., Richter G., Weber S., On the Reaction Mechanism of Adduct Formation in LOV Domains of the Plant Blue-Light Receptor Phototropin. J. Am. Chem. Soc. 126, 11067–11076 (2004). 15339193 10.1021/ja049553q

[17] Christie J. M., Reymond P., Powell G. K., Bernasconi P., Raibekas A. A., Liscum E., Briggs W. R., Arabidopsis NPH1: A Flavoprotein with the Properties of a Photoreceptor for Phototropism. Science 282, 1698–1701 (1998). 9831559 10.1126/science.282.5394.1698

[18] Halavaty A. S., Moffat K., N- and C-Terminal Flanking Regions Modulate Light-Induced Signal Transduction in the LOV2 Domain of the Blue Light Sensor Phototropin 1 from Avena sativa,. Biochemistry 46, 14001–14009 (2007). 18001137 10.1021/bi701543e

[19] Alexandre M. T. A., van Grondelle R., Hellingwerf K. J., Kennis J. T. M., Conformational Heterogeneity and Propagation of Structural Changes in the LOV2/Jα Domain from Avena sativa Phototropin 1 as Recorded by Temperature-Dependent FTIR Spectroscopy. Biophysical Journal 97, 238–247 (2009). 19580761 10.1016/j.bpj.2009.03.047 PMC2711381

[20] Harper S. M., Neil L. C., Day I. J., Hore P. J., Gardner K. H., Conformational Changes in a Photosensory LOV Domain Monitored by Time-Resolved NMR Spectroscopy. J. Am. Chem. Soc. 126, 3390–3391 (2004). 15025443 10.1021/ja038224f

[21] Maity S., Price B. D., Wilson C. B., Mukherjee A., Starck M., Parker D., Wilson M. Z., Lovett J. E., Han S., Sherwin M. S., Triggered Functional Dynamics of AsLOV2 by Time-Resolved Electron Paramagnetic Resonance at High Magnetic Fields. Angewandte Chemie International Edition 62, e202212832 (2023). 36638360 10.1002/anie.202212832 PMC12459748

[22] Harper S. M., Neil L. C., Gardner K. H., Structural Basis of a Phototropin Light Switch. Science 301, 1541–1544 (2003). 12970567 10.1126/science.1086810

[23] Zayner J. P., Antoniou C., Sosnick T. R., The Amino-Terminal Helix Modulates Light-Activated Conformational Changes in AsLOV2. Journal of Molecular Biology 419, 61–74 (2012). 22406525 10.1016/j.jmb.2012.02.037 PMC3338903

[24] Harper S. M., Christie J. M., Gardner K. H., Disruption of the LOV–Jα Helix Interaction Activates Phototropin Kinase Activity. Biochemistry 43, 16184–16192 (2004). 15610012 10.1021/bi048092i

[25] Freddolino P. L., Gardner K. H., Schulten K., Signaling mechanisms of LOV domains: New insights from molecular dynamics studies. Photochem Photobiol Sci 12, 1158–1170 (2013). 23407663 10.1039/c3pp25400c PMC3679247

[26] Song S.-H., Freddolino P. L., Nash A. I., Carroll E. C., Schulten K., Gardner K. H., Larsen D. S., Modulating LOV Domain Photodynamics with a Residue Alteration outside the Chromophore Binding Site. Biochemistry 50, 2411–2423 (2011). 21323358 10.1021/bi200198x PMC3068209

[27] Alexandre M. T. A., Arents J. C., van Grondelle R., Hellingwerf K. J., Kennis J. T. M., A Base-Catalyzed Mechanism for Dark State Recovery in the Avena sativa Phototropin-1 LOV2 Domain. Biochemistry 46, 3129–3137 (2007). 17311415 10.1021/bi062074e

[28] Zayner J. P., Sosnick T. R., Factors That Control the Chemistry of the LOV Domain Photocycle. PLoS One 9, e87074 (2014). 24475227 10.1371/journal.pone.0087074 PMC3903614

[29] Chan R. H., Bogomolni R. A., Structural Water Cluster As a Possible Proton Acceptor in the Adduct Decay Reaction of Oat Phototropin 1 LOV2 Domain. J. Phys. Chem. B 116, 10609–10616 (2012). 22845056 10.1021/jp304934t

[30] Zoltowski B. D., Vaccaro B., Crane B. R., Mechanism-based tuning of a LOV domain photoreceptor. Nat Chem Biol 5, 827–834 (2009). 19718042 10.1038/nchembio.210 PMC2865183

[31] Yang J., Wang Y., Wang L., Zhong D., Mapping Hydration Dynamics around a β-Barrel Protein. J. Am. Chem. Soc. 139, 4399–4408 (2017). 28248506 10.1021/jacs.6b12463

[32] Zaragoza J. P. T., Nguy A., Minnetian N., Deng Z., Iavarone A. T., Offenbacher A. R., Klinman J. P., Detecting and Characterizing the Kinetic Activation of Thermal Networks in Proteins: Thermal Transfer from a Distal, Solvent-Exposed Loop to the Active Site in Soybean Lipoxygenase. J. Phys. Chem. B 123, 8662–8674 (2019). 31580070 10.1021/acs.jpcb.9b07228 PMC6944211

[33] Qin Y., Wang L., Zhong D., Dynamics and mechanism of ultrafast water–protein interactions. Proceedings of the National Academy of Sciences 113, 8424–8429 (2016). 10.1073/pnas.1602916113 PMC4968723 27339138

[34] Qin Y., Jia M., Yang J., Wang D., Wang L., Xu J., Zhong D., Molecular Origin of Ultrafast Water–Protein Coupled Interactions. J. Phys. Chem. Lett. 7, 4171–4177 (2016). 27700094 10.1021/acs.jpclett.6b01954

[35] Kim S. B., Gupta D. R., Debenedetti P. G., Computational investigation of dynamical transitions in Trp-cage miniprotein powders. Sci Rep 6, 25612 (2016). 27151767 10.1038/srep25612 PMC4858699

[36] Mukherjee S., Ramos S., Pezzotti S., Kalarikkal A., Prass T. M., Galazzo L., Gendreizig D., Barbosa N., Bordignon E., Havenith M., Schäfer L. V., Entropy Tug-of-War Determines Solvent Effects in the Liquid–Liquid Phase Separation of a Globular Protein. J. Phys. Chem. Lett. 15, 4047–4055 (2024). 38580324 10.1021/acs.jpclett.3c03421 PMC11033941

[37] Ramos S., Kamps J., Pezzotti S., Winklhofer K. F., Tatzelt J., Havenith M., Hydration makes a difference! How to tune protein complexes between liquid–liquid and liquid–solid phase separation. Physical Chemistry Chemical Physics 25, 28063–28069 (2023). 37840355 10.1039/d3cp03299j

[38] Conti Nibali V., Pezzotti S., Sebastiani F., Galimberti D. R., Schwaab G., Heyden M., Gaigeot M.-P., Havenith M., Wrapping Up Hydrophobic Hydration: Locality Matters. J. Phys. Chem. Lett. 11, 4809–4816 (2020). 32459100 10.1021/acs.jpclett.0c00846 PMC8253475

[39] Mahanta D. D., Brown D. R., Webber T., Pezzotti S., Schwaab G., Han S., Shell M. S., Havenith M., Bridging the Gap in Cryopreservation Mechanism: Unraveling the Interplay between Structure, Dynamics, and Thermodynamics in Cryoprotectant Aqueous Solutions. J. Phys. Chem. B 128, 3720–3731 (2024). 38584393 10.1021/acs.jpcb.4c00264

[40] Pezzotti S., Chen W., Novelli F., Yu X., Hoberg C., Havenith M., Terahertz calorimetry spotlights the role of water in biological processes. Nat Rev Chem, 1–14 (2025). 40346278 10.1038/s41570-025-00712-8

[41] Pezzotti S., Sebastiani F., van Dam E. P., Ramos S., Conti Nibali V., Schwaab G., Havenith M., Spectroscopic Fingerprints of Cavity Formation and Solute Insertion as a Measure of Hydration Entropic Loss and Enthalpic Gain. Angewandte Chemie International Edition 61, e202203893 (2022). 35500074 10.1002/anie.202203893 PMC9401576

[42] Mahanta D. D., Brown D. R., Pezzotti S., Han S., Schwaab G., Shell M. S., Havenith M., Local solvation structures govern the mixing thermodynamics of glycerol–water solutions. Chem. Sci. 14, 7381–7392 (2023). 37416713 10.1039/d3sc00517h PMC10321518

[43] Pezzotti S., König B., Ramos S., Schwaab G., Havenith M., Liquid–Liquid Phase Separation? Ask the Water! J. Phys. Chem. Lett. 14, 1556–1563 (2023). 36745512 10.1021/acs.jpclett.2c02697

[44] Glantz S. T., Carpenter E. J., Melkonian M., Gardner K. H., Boyden E. S., Wong G. K.-S., Chow B. Y., Functional and topological diversity of LOV domain photoreceptors. Proceedings of the National Academy of Sciences 113, E1442–E1451 (2016). 10.1073/pnas.1509428113 PMC4801262 26929367

[45] Swartz T. E., Tseng T.-S., Frederickson M. A., Paris G., Comerci D. J., Rajashekara G., Kim J.-G., Mudgett M. B., Splitter G. A., Ugalde R. A., Goldbaum F. A., Briggs W. R., Bogomolni R. A., Blue-Light-Activated Histidine Kinases: Two-Component Sensors in Bacteria. Science 317, 1090–1093 (2007). 17717187 10.1126/science.1144306

[46] Zoltowski B. D., Schwerdtfeger C., Widom J., Loros J. J., Bilwes A. M., Dunlap J. C., Crane B. R., Conformational Switching in the Fungal Light Sensor Vivid. Science 316, 1054–1057 (2007). 17510367 10.1126/science.1137128 PMC3682417

[47] Yee E. F., Diensthuber R. P., Vaidya A. T., Borbat P. P., Engelhard C., Freed J. H., Bittl R., Möglich A., Crane B. R., Signal transduction in light–oxygen–voltage receptors lacking the adduct-forming cysteine residue. Nat Commun 6, 10079 (2015). 26648256 10.1038/ncomms10079 PMC4682037

[48] Zayner J. P., Antoniou C., French A. R., Hause R. J., Sosnick T. R., Investigating Models of Protein Function and Allostery With a Widespread Mutational Analysis of a Light-Activated Protein. Biophysical Journal 105, 1027–1036 (2013). 23972854 10.1016/j.bpj.2013.07.010 PMC3752136

[49] Iuliano J. N., Collado J. T., Gil A. A., Ravindran P. T., Lukacs A., Shin S., Woroniecka H. A., Adamczyk K., Aramini J. M., Edupuganti U. R., Hall C. R., Greetham G. M., Sazanovich I. V., Clark I. P., Daryaee T., Toettcher J. E., French J. B., Gardner K. H., Simmerling C. L., Meech S. R., Tonge P. J., Unraveling the Mechanism of a LOV Domain Optogenetic Sensor: A Glutamine Lever Induces Unfolding of the Jα Helix. ACS Chem. Biol. 15, 2752–2765 (2020). 32880430 10.1021/acschembio.0c00543 PMC7572778

[50] Price B. D., Sojka A., Maity S., Chavez I. M., Starck M., Wilson M. Z., Han S., Sherwin M. S., Field-domain rapid-scan EPR at 240 GHz for studies of protein functional dynamics at room temperature. Journal of Magnetic Resonance 366, 107744 (2024). 39096714 10.1016/j.jmr.2024.107744 PMC12459749

[51] Schiemann O., Heubach C. A., Abdullin D., Ackermann K., Azarkh M., Bagryanskaya E. G., Drescher M., Endeward B., Freed J. H., Galazzo L., Goldfarb D., Hett T., Esteban Hofer L., Fábregas Ibáñez L., Hustedt E. J., Kucher S., Kuprov I., Lovett J. E., Meyer A., Ruthstein S., Saxena S., Stoll S., Timmel C. R., Di Valentin M., Mchaourab H. S., Prisner T. F., Bode B. E., Bordignon E., Bennati M., Jeschke G., Benchmark Test and Guidelines for DEER/PELDOR Experiments on Nitroxide-Labeled Biomolecules. J. Am. Chem. Soc. 143, 17875–17890 (2021). 34664948 10.1021/jacs.1c07371 PMC11253894

[52] Milov A.D., Salikhov K. M., Shirov M. D., Application of the double resonance method to electron spin echo in a study of the spatial of the spatial distribution of paramagnetic centers in solids. Fiz. Tverd. Tela 23, 975–982 (1981).

[53] Milov A. D., Ponomarev A. B., Tsvetkov Yu. D., Electron-electron double resonance in electron spin echo: Model biradical systems and the sensitized photolysis of decalin. Chemical Physics Letters 110, 67–72 (1984).

[54] Martin R. E., Pannier M., Diederich F., Gramlich V., Hubrich M., Spiess H. W., Determination of End-to-End Distances in a Series of TEMPO Diradicals of up to 2.8 nm Length with a New Four-Pulse Double Electron Electron Resonance Experiment. Angewandte Chemie International Edition 37, 2833–2837 (1998). 29711097 10.1002/(SICI)1521-3773(19981102)37:20<2833::AID-ANIE2833>3.0.CO;2-7

[55] Armstrong B. D., Han S., Overhauser Dynamic Nuclear Polarization To Study Local Water Dynamics. J. Am. Chem. Soc. 131, 4641–4647 (2009). 19290661 10.1021/ja809259q

[56] Franck J. M., Han S., “Chapter Five - Overhauser Dynamic Nuclear Polarization for the Study of Hydration Dynamics, Explained” in Methods in Enzymology, Wand A. J., Ed. (Academic Press, 2019; https://www.sciencedirect.com/science/article/pii/S0076687918303872)vol. 615 of Biological NMR Part B, pp. 131–175. 30638529 10.1016/bs.mie.2018.09.024

[57] Franck J. M., Pavlova A., Scott J. A., Han S., Quantitative cw Overhauser effect dynamic nuclear polarization for the analysis of local water dynamics. Progress in Nuclear Magnetic Resonance Spectroscopy 74, 33–56 (2013). 24083461 10.1016/j.pnmrs.2013.06.001 PMC3798041

[58] Pavlova A., Cheng C.-Y., Kinnebrew M., Lew J., Dahlquist F. W., Han S., Protein structural and surface water rearrangement constitute major events in the earliest aggregation stages of tau. Proceedings of the National Academy of Sciences 113, E127–E136 (2016). 10.1073/pnas.1504415113 PMC4720316 26712030

[59] Hussain S., Franck J. M., Han S., Transmembrane Protein Activation Refined by Site-Specific Hydration Dynamics. Angewandte Chemie International Edition 52, 1953–1958 (2013). 23307344 10.1002/anie.201206147

[60] Vigers M. P., Lobo S., Najafi S., Dubose A., Tsay K., Ganguly P., Longhini A. P., Jin Y., Buratto S. K., Kosik K. S., Shell M. S., Shea J.-E., Han S., Water-directed pinning is key to tau prion formation. Proceedings of the National Academy of Sciences 122, e2421391122 (2025). 10.1073/pnas.2421391122 PMC12067210 40294272

[61] Konold P. E., Mathes T., Weiβenborn J., Groot M. L., Hegemann P., Kennis J. T. M., Unfolding of the C-Terminal Jα Helix in the LOV2 Photoreceptor Domain Observed by Time-Resolved Vibrational Spectroscopy. J. Phys. Chem. Lett. 7, 3472–3476 (2016). 27537211 10.1021/acs.jpclett.6b01484

[62] Bank R. P. D., RCSB PDB - 2V1A: N- and C-terminal helices of oat LOV2 (404–546) are involved in light-induced signal transduction (room temperature (293K) dark structure of LOV2 (404–546)). https://www.rcsb.org/structure/2V1A.

[63] Ravula T., Sahoo B. R., Dai X., Ramamoorthy A., Natural-abundance 17 O NMR spectroscopy of magnetically aligned lipid nanodiscs. doi: 10.1039/D0CC04011H (2020). PMC7484029 32724998

[64] Keeler E. G., Michaelis V. K., Wilson C. B., Hung I., Wang X., Gan Z., Griffin R. G., High-Resolution 17O NMR Spectroscopy of Structural Water. J. Phys. Chem. B 123, 3061–3067 (2019). 30882222 10.1021/acs.jpcb.9b02277 PMC6689193

[65] Keeler E. G., Michaelis V. K., Griffin R. G., 17O NMR Investigation of Water Structure and Dynamics. J. Phys. Chem. B 120, 7851–7858 (2016). 27454747 10.1021/acs.jpcb.6b05755 PMC5418864

[66] Michaelis V. K., Keeler E. G., Ong T.-C., Craigen K. N., Penzel S., Wren J. E. C., Kroeker S., Griffin R. G., Structural Insights into Bound Water in Crystalline Amino Acids: Experimental and Theoretical 17O NMR. J. Phys. Chem. B 119, 8024–8036 (2015). 25996165 10.1021/acs.jpcb.5b04647 PMC4894719

[67] Paulino J., Yi M., Hung I., Gan Z., Wang X., Chekmenev E. Y., Zhou H.-X., Cross T. A., Functional stability of water wire–carbonyl interactions in an ion channel. Proceedings of the National Academy of Sciences 117, 11908–11915 (2020). 10.1073/pnas.2001083117 PMC7275723 32414918

[68] Krivdin L. B., 17O nuclear magnetic resonance: Recent advances and applications. Magnetic Resonance in Chemistry 61, 507–529 (2023). 37449419 10.1002/mrc.5378

[69] Fábregas Ibáñez L., Jeschke G., Stoll S., DeerLab: a comprehensive software package for analyzing dipolar electron paramagnetic resonance spectroscopy data. Magnetic Resonance 1, 209–224 (2020). 34568875 10.5194/mr-1-209-2020 PMC8462493

[70] Zhang R., Cross T. A., Peng X., Fu R., Surprising Rigidity of Functionally Important Water Molecules Buried in the Lipid Headgroup Region. J. Am. Chem. Soc. 144, 7881–7888 (2022). 35439409 10.1021/jacs.2c02145 PMC9165019

[71] Potnuru L.R., Najafi S., Webber T., Brown D., Chaklashiya R., Jameson CJ, de Dios AC, Han S., 17O NMR Chemical Shift Reports on Water Structural Property in DMSO-Water Mixtures (manuscript in preparation).

[72] Akasaka K., Li H., Yamada H., Li R., Thoresen T., Woodward C. K., Pressure response of protein backbone structure. Pressure-induced amide 15N chemical shifts in BPTI. Protein Science 8, 1946–1953 (1999). 10548039 10.1110/ps.8.10.1946 PMC2144150

[73] Kitahara R., Hata K., Li H., Williamson M. P., Akasaka K., Pressure-induced chemical shifts as probes for conformational fluctuations in proteins. Progress in Nuclear Magnetic Resonance Spectroscopy 71, 35–58 (2013). 23611314 10.1016/j.pnmrs.2012.12.001

[74] Akasaka K., Probing Conformational Fluctuation of Proteins by Pressure Perturbation. Chem. Rev. 106, 1814–1835 (2006). 16683756 10.1021/cr040440z

[75] Akasaka K., Li H., Low-Lying Excited States of Proteins Revealed from Nonlinear Pressure Shifts in 1H and 15N NMR. Biochemistry 40, 8665–8671 (2001). 11467925 10.1021/bi010312u

[76] Akasaka K., Exploring the entire conformational space of proteins by high-pressure NMR. Pure and Applied Chemistry 75, 927–936 (2003).

[77] Russell H., “Under pressure : measuring nanometre distance changes in biomolecules,” thesis, The University of St Andrews (2024).

[78] Lerch M. T., Yang Z., Altenbach C., Hubbell W. L., “Chapter Two - High-Pressure EPR and Site-Directed Spin Labeling for Mapping Molecular Flexibility in Proteins” in Methods in Enzymology, Qin P. Z., Warncke K., Eds. (Academic Press, 2015; https://www.sciencedirect.com/science/article/pii/S0076687915004085)vol. 564 of Electron Paramagnetic Resonance Investigations of Biological Systems by Using Spin Labels, Spin Probes, and Intrinsic Metal Ions, Part B, pp. 29–57. 26477247 10.1016/bs.mie.2015.07.004

[79] Lerch M. T., Yang Z., Brooks E. K., Hubbell W. L., Mapping protein conformational heterogeneity under pressure with site-directed spin labeling and double electron–electron resonance. Proceedings of the National Academy of Sciences 111, E1201–E1210 (2014). 10.1073/pnas.1403179111 PMC3977274 24707053

[80] Lerch M. T., López C. J., Yang Z., Kreitman M. J., Horwitz J., Hubbell W. L., Structure-relaxation mechanism for the response of T4 lysozyme cavity mutants to hydrostatic pressure. Proceedings of the National Academy of Sciences 112, E2437–E2446 (2015). 10.1073/pnas.1506505112 PMC4434698 25918400

[81] Lerch M. T., Matt R. A., Masureel M., Elgeti M., Kumar K. K., Hilger D., Foys B., Kobilka B. K., Hubbell W. L., Viewing rare conformations of the β2 adrenergic receptor with pressure-resolved DEER spectroscopy. Proceedings of the National Academy of Sciences 117, 31824–31831 (2020). 10.1073/pnas.2013904117 PMC7749303 33257561

[82] Arsiccio A., Shea J.-E., Pressure Unfolding of Proteins: New Insights into the Role of Bound Water. J. Phys. Chem. B 125, 8431–8442 (2021). 34310136 10.1021/acs.jpcb.1c04398

[83] Monroe J. I., Shell M. S., Decoding signatures of structure, bulk thermodynamics, and solvation in three-body angle distributions of rigid water models. The Journal of Chemical Physics 151, 094501 (2019). 31492058 10.1063/1.5111545

[84] Najafi S., Lobo S., Shell M. S., Shea J.-E., Context Dependency of Hydrophobicity in Intrinsically Disordered Proteins: Insights from a New Dewetting Free Energy-Based Hydrophobicity Scale. J. Phys. Chem. B 129, 1904–1915 (2025). 39907269 10.1021/acs.jpcb.4c06399 PMC11848916

[85] MacKerell A. D. Jr., Bashford D., Bellott M., Dunbrack R. L. Jr., Evanseck J. D., Field M. J., Fischer S., Gao J., Guo H., Ha S., Joseph-McCarthy D., Kuchnir L., Kuczera K., Lau F. T. K., Mattos C., Michnick S., Ngo T., Nguyen D. T., Prodhom B., Reiher W. E., Roux B., Schlenkrich M., Smith J. C., Stote R., Straub J., Watanabe M., Wiórkiewicz-Kuczera J., Yin D., Karplus M., All-Atom Empirical Potential for Molecular Modeling and Dynamics Studies of Proteins. J. Phys. Chem. B 102, 3586–3616 (1998). 24889800 10.1021/jp973084f

[86] Daura X., Gademann K., Jaun B., Seebach D., van Gunsteren W. F., Mark A. E., Peptide Folding: When Simulation Meets Experiment. Angewandte Chemie International Edition 38, 236–240 (1999).

[87] Abraham M. J., Murtola T., Schulz R., Páll S., Smith J. C., Hess B., Lindahl E., GROMACS: High performance molecular simulations through multi-level parallelism from laptops to supercomputers. SoftwareX 1–2, 19–25 (2015).

[88] Truong C., Oudre L., Vayatis N., Selective review of offline change point detection methods. Signal Processing 167, 107299 (2020).

[89] Yao X., Rosen M. K., Gardner K. H., Estimation of the available free energy in a LOV2-Jα photoswitch. Nat Chem Biol 4, 491–497 (2008). 18604202 10.1038/nchembio.99 PMC2597337

[90] Strickland D., Yao X., Gawlak G., Rosen M. K., Gardner K. H., Sosnick T. R., Rationally improving LOV domain–based photoswitches. Nat Methods 7, 623–626 (2010). 20562867 10.1038/nmeth.1473 PMC2914111

[91] Hoffmann K. H., Kroell A.-S., Motzkus N. A., Lemmen N., Happ N., Wolf B., von Bachmann A., Southern N., Vogd F., Aschenbrenner S., Niopek D., Mathony J., Modular Engineering of Thermo-Responsive Allosteric Proteins. bioRxiv [Preprint] (2025). 10.1101/2025.05.02.651844. PMC13128441 41680487

